# Quality of life: predictors and outcomes after stroke in a Brazilian public hospital

**DOI:** 10.1055/s-0042-1758364

**Published:** 2023-03-14

**Authors:** Camila Thieime Rosa, Marise Bueno Zonta, Marcos Christiano Lange, Viviane de Hiroki Flumignam Zétola

**Affiliations:** 1 Universidade Federal do Paraná, Hospital de Clínicas, Unidade de AVC, Departamento de Clínica Médica, Curitiba PR, Brazil.

**Keywords:** Stroke, Quality of Life, Stroke Rehabilitation, Health Services Accessibility, Patient Reported Outcome Measures, Acidente Vascular Cerebral, Qualidade de Vida, Reabilitação do Acidente Vascular Cerebral, Acesso aos Serviços de Saúde, Medidas de Resultados Relatados pelo Paciente

## Abstract

**Background** Some scales are applied after stroke to measure functional independence but qualify of life (QoL) is sometimes neglected in this scenario.

**Objective** To analyze predictors and outcomes of QoL after stroke using a validated scale in our population.

**Methods** Our study included patients who had their first ischemic stroke and were followed in the outpatient clinic for at least 6 months from stroke index. Disability status was assessed using the modified Rankin scale (mRS), the Barthel index (BI), and the Lawton and Brody scale. Quality of life was assessed by a stroke-specific QoL (SSQoL) scale. Statistical significance was accepted for *p* < 0.05. The estimated measure of association was the odds ratio (OR) for which 95% confidence intervals (95%Cis) were presented.

**Results** Of 196 patients studied, the median age was 60.4 (±13.4) years, and 89 (45.40%) of the patients were female. In a stepwise model considering risk factors, basic activities of daily living scales, satisfaction with life, and outcomes, we found four independent variables related to a poor QoL after stroke, namely hypertension, non-regular rehabilitation, not returning to work, and medical complications. The National Institutes of Health stroke scale (NIHSS) score at admission ≥ 9 was also an independent clinical marker. Approximately 30% of all participants had a negative score under 147 points in the SSQoL.

**Conclusions** Our results showed that QoL after stroke in a developing country did not seem to differ from those of other countries, although there is a gap in rehabilitation programs in our public system. The functional scales are important tools, but they have failed to predict QoL, in some patients, when compared with specific scales.

## Resumo

**Antecedentes** Algumas escalas são aplicadas após o acidente vascular cerebral (AVC) para avaliar a independência funcional, mas a qualidade de vida (QV) às vezes é negligenciada nesse cenário.

**Objetivo** Analisar preditores e desfechos de QV após AVC utilizando uma escala validada em nossa população.

**Métodos** Nosso estudo incluiu pacientes que tiveram seu primeiro AVC isquêmico e foram acompanhados no ambulatório por pelo menos 6 meses após o AVC. A funcionalidade foi avaliada pela escala de Rankin modificada, índice de Barthel e escala de Lawton e Brody. A QV foi avaliada pela ecala de qualidade de vida específica de acidente vascular cerebral (SSQoL). A significância estatística aceita foi *p* < 0,05. A medida de associação estimada foi o *odds ratio* (OR), para o qual foram apresentados intervalos de confiança (IC) de 95%.

**Resultados** Dos 196 pacientes, a média de idade foi de 60,4 (±13,4) anos, sendo 89 (45,40%) do sexo feminino. Em um modelo *stepwise* considerando fatores de risco, escalas de atividades básicas da vida diária, satisfação com a vida e desfechos, encontramos quatro variáveis independentes relacionadas a uma QV ruim após o AVC, como hipertensão, reabilitação não regular, não retorno ao trabalho e comorbidades pós-AVC. A pontuação NIHSS na admissão ≥ 9 também foi um marcador clínico independente. Aproximadamente 30% de todos os participantes tiveram uma pontuação abaixo de 147 pontos para SSQoL.

**Conclusões** Nossos resultados mostraram que a QV após AVC em um país em desenvolvimento não parece diferir de outros países, apesar da lacuna nos programas de reabilitação em nosso sistema público. As escalas funcionais são ferramentas importantes, mas falharam em alguns pacientes em predizer a QV quando comparadas com escalas específicas.

## INTRODUCTION

Stroke is one of the main causes of death and the first cause of disability worldwide, 1 impairing activities of daily life and, consequently, quality of life (QoL). 2 3 The outcomes of stroke seem to correlate with predictors related to QoL in stroke patients, 4 5 such as age, 2 6 sex, 2 7 8 economic status, 9 10 employment, educational level, 11 marital status, ability to return to work, 12 13 presence of daily caregiver, 8 14 medical complications after stroke, 8 15 16 and regular rehabilitation and functional scales. 17 All these findings can contribute to the development of public policies which aim to improve care among stroke survivors. However, there are only a few studies that evaluate long-term QoL after stroke in a developing country.

Due to the high prevalence of cerebrovascular disease in Brazil, a *Stroke Care Line* was developed by the Brazilian Stroke Society together with the Ministry of Health. 18 This protocol starts with primary care in a basic health network involving population campaigns to recognize the signs and symptoms of stroke, including training the emergency medical service (EMS), called SAMU, and a Comprehensive Stroke Center with hospital management protocols, neuroimaging, and investigation exams for appropriate secondary prevention. We also have some complimentary medicine delivered, such as antiplatelet, statins, antihypertensives, and diabetes treatment. There are currently 67 certified high-quality centers in Brazil, not too much for a large country, but new efforts to get a Latin American certification have started. Due to the lack of specialized physicians, some tele stroke programs have also been organized to provide online assistance. However, we all know that acute treatment does not always allow independence in these patients, and rehabilitation centers are an important method to reinsert the patient into a productive society and reduce the social burden. Besides that, QoL seems to have a high contribution to improve in all means of the patient life, including mental health, and guiding rehabilitation therapy. Unfortunately, in our public health system, it is still missing in the stroke care line. There is no real data about how the patient reaches a specialized team and how it influences the daily functional abilities. Standards of stroke outcome tools can be used to evaluate individual patients, such as the modified Rankin scale (mRS), Barthel, but it does not necessarily identify all predictors of QoL. Most likely, in a developing country such as Brazil, differences in care can be found based on economic and social levels. 3 14

The present study aimed to measure QoL after the first stroke in patients attended at a Brazilian public hospital. We also investigated the association between predictors and outcomes in this population.

## METHODS

Our study included patients who had experienced their first ischemic stroke. All were admitted to a public stroke center and were followed in an outpatient clinic for at least 6 months from stroke index. We interview all stroke patients at the outpatient clinic for 1 year and selected them according to inclusion and exclusion criteria. They were interviewed between June 2016 and July 2017. This study was conducted ethically following the local ethics committee (CAAE 34716314.7.0000.0096). Written and oral informed consent was obtained from the study participants.

Patients were eligible to participate in the present study if they were 18 years or older and had received neuroimaging (computed tomography [CT] scan or magnetic resonance imaging [MRI]) during admission to confirm the presence of a brain lesion. The exclusion criteria were refusal to participate, prior clinical stroke, recurrence of stroke during the study period, cognitive impairment, and aphasia based on medical records. Stroke was classified based on the Trial of Org 10172 in Acute Stroke Treatment classification system (TOAST) criteria. 19

Of the 305 patients admitted with their first ischemic stroke during the study period, 16 (5.24%) refused to participate, 45 (14.75%) presented with cognitive impairment, and 48 (15.75%) died. A total of 196 (64.26%) participants comprises the analytic cohort. We collected sociodemographic and clinical data—including scores for the National Institutes of Health stroke scale (NIHSS)—at hospital admission and scores of dependences at discharge. We analyzed the results from the Barthel index (BI) and the modified Rankin scale (mRS) to measure basic activities of daily living (BADL), 20 21 and the Lawton and Brody inventory to measure instrumental activities of daily living (IADL) on the same day of the interview. 22 Individuals were considered independent if they had a BI score > 60 and a mRS ≤ 2 20 21 for BADL and a score equal to 21 22 for IADL. A stroke-specific quality of life (SSQoL) scale was applied to assess QoL. 23 The investigator performed all measures. The SSQoL consists of multiple health domains using patient-reported outcomes (PROs). This scale has 49 questions that assess the following 12 domains: mobility, energy, upper extremity function, work/productivity, mood, self-care, social roles, family roles, vision, thinking, personality, and language. Scores range from 49 (very poor QoL) to 245 (optimal QoL). Values below or equal to 147 points (cut-off) were considered as poor QoL. 11 14 24 25 This scale is validated in Portuguese for a Brazilian population. 26

Statistical analyses were performed using Stata/SE v.14.1 software (Stata Corp LLC, College Station, TX, USA). For comparison of quantitative variables, the Student *t* -test and the Mann-Whitney U or Kruskal-Wallis tests were conducted, considering normal distribution. Categorical variables were analyzed using a Chi-squared test or Fisher exact test. The normality of data was determined using the Kolmogorov-Smirnov test. The Spearman correlation coefficient was considered to analyze correlations between quantitative variables. For multivariable analysis, factors associated with a poor QoL, logistic regression models were adjusted, considering a stepwise backward approach 27 and including variables with *p* < 0.25 in the univariate analysis. The estimated measure of association was the odds ratio (OR) for which 95% confidence intervals (95%ICs) were presented. There was no evidence of inadequate adjustments. Statistical significance was accepted for *p* < 0.05.

## RESULTS

Of the 196 patients, the median age was 60.4 (±13.4) years, and 89 (45.40%) of the patients were female. The mean time from stroke index was 20 (±13) months. Significant risk factors were hypertension in 42 (70%; *p* = 0.041) and diabetes in 22 (37%; *p* = 0.021) patients. Stroke-specific quality of life scores showed that 60 (31.6%) participants had poor QoL. A NIHSS score ≥ 9 or more on admission was correlated with poor QoL ( *p* = 0.001). Although 25 (42%) patients had been classified with a good BADL score (mRS ≤ 2), they also rated a poor QoL ( *p* < 0.01). The demographic data and vascular risk factors are presented in Table 1 .

**Table 1:** Demographic data and risk factors in patients with first ischemic stroke (before admission)

Variable		a Poor QoL* n (%) [ *n* = 60]	Good QoL* N (%) [ *n* = 136]	Univariate *p* -value
Mean age		60.9 ± 12.2	60.3 ± 14.2	0.809
Female sex		31 (52)	58 (43)	0.277
Hypertension		42 (70)	73 (54)	0.041*
Diabetes mellitus		22 (37)	28 (21)	0.021*
Hypercholesterolemia		14 (23)	31 (23)	1
Smoking		20 (33)	33 (24)	0.222
Alcohol consumption		12 (20)	14 (10)	0.072
Atrial fibrillation		1 (2)	9 (7)	0.288
Coronary artery disease		7 (12)	14 (10)	0.804
Peripheral artery disease		0 (0)	3 (2)	0.554
Marital status	Married	40 (67)	92 (68)	0.784
	Single	4 (7)	11 (8)	
	Divorced	8 (13)	12 (9)	
	Widower	8 (13)	21 (15)	
Educational level	Illiterate	3 (5)	6 (4)	0.232
	1–4 years	24 (40)	37 (27)	
	5–8 years	11 (18)	37 (27)	
	9–11 years	19 (32)	40 (30)	
	≥ 12 years	3 (5)	16 (12)	
b Economic status	≤ 1 salary	14 (23)	24 (18)	0.455
	2–4 salaries	38 (64)	86 (63)	
	5–7 salaries	4 (7)	19 (14)	
	≥ 8 salaries	2 (3)	4 (3)	
	NA	2 (3)	3 (2)	

Notes: *Student *t* -test and Mann-Whitney U or Kruskal-Wallis; Chi-squared test or Fisher exact test; *p* <0.05. ^a^ Quality of life with less than 147 points in the SSQOL scale; ^b^ Economic status (minimum monthly wage provided for by law R$ 954.00 = US$ 253.05 [US dollar quoted at R$ 3.77]).

Because the interview occurs at least 6 months after the discharge, new variables were assessed: the presence of a daily caregiver ( *p* < 0.001), not returning to work ( *p* = 0.001), and irregular rehabilitation ( *p* = 0.007). All of them were significant. At the time of the interview, both BADL and IADL were correlating with poor QoL ( Table 2 ) ( Table 3 ).

**Table 2:** Clinical Characteristics in patients with stroke and medical complications

Variable		a Poor QoL* n (%) [ *n* = 60]	Good QoL* N (%) [ *n* = 136]	Univariate *p* -value
NIHSS at admission (min–max)		9 (0–21)	5 (0–20)	< 0.001*
Stroke side	Left	23 (38)	73 (54)	0.229
	Right	31 (52)	53 (39)	
	Brainstem	5 (8)	7 (5)	
	Bilateral hemisphere	1 (2)	3 (2)	
Thrombolysis		32 (53)	52 (38)	0.060
TOAST classification	Cardioembolism	14 (23)	37 (27)	0.745
	Large artery atherosclerosis	16 (27)	25 (18)	
	Undetermined etiology	19 (32)	44 (33)	
	Other determined etiologies	3 (5)	7 (5)	
	Small vessels occlusion	8 (13)	23 (17)	
Medical complications after stroke		28 (47)	45 (33)	0.079

Abbreviation: NIHSS, National Institutes of Health Stroke Scale; QoL, quality of life.

Notes: *Student *t* -test and Mann-Whitney U or Kruskal-Wallis; Chi-squared test or Fisher exact test; *p* <0.05; ^a^ Quality of life with less than 147 points in the SSQOL scale.

**Table 3:** Outpatients assessment

Variable		a Poor QoL n (%) [ *n* = 60]	Good QoL N (%) [ *n* = 136]	Univariate *p* -value
Daily caregiver		23 (88.5)	3 (11.5)	< 0.001*
Not returned to work		35 (58)	34 (25)	< 0.001*
No regular rehabilitation		23 (38)	26 (19)	0.007*
At interview	mRS ≤ 2 at interview	33 (55)	130 (96)	< 0.001*
	Barthel index ≥ 60	29 (48)	125 (92)	< 0.001*
	Lawton and Brody ≥ 21	6 (10)	78 (57)	< 0.001*

Abbreviations: BADL, basic activities daily of living; IADL, Instrumental activities of daily living; mRS, modified Rankin score.

Notes: *Student *t* -test and Mann-Whitney U or Kruskal-Wallis; Chi-squared test or Fisher exact test; *p* <0.05; ^a^ Quality of Life with less than 147 points in the SSQOL scale.

In the multivariable analysis, hypertension, irregular rehabilitation, unemployment, medical complications after stroke, and dependence on BADL and IADL were also predictors of poor QoL ( Table 4 ).

**Table 4:** Multivariate analysis

Variable	Odds ratio (CI 95%)	Multivariate *p* -value*
Hypertension	2.68 (1.38–14.9)	0.027*
Not returned to work	2.61 (1.13–6.04)	0.025*
No regular rehabilitation	3.31 (1.39–7.92)	0.007*
Medical complications after stroke	2.34 (1.01–5.42)	0.046*
mRS ≤ 2 at interview	7.69 (1.85–32.0)	0.005*
Barthel index ≥ 60	4.53 (1.38–14.9)	0.013*

Abbreviations: CI, confidence interval; mRS, modified Rankin score; BADL, basic activities daily of living; IADL, instrumental activities of daily living.

Notes: When analyze Lawton e Brody ≥ 21: <0.001* 7.53 (2.76–20.5); *Multivariate Logistic Regression Model (stepwise backward) and Wald test, *p* <0.05; ^a^ Quality of Life with less than 147 points in the SSQOL scale.

We also analyzed scores for each SSQoL domain. Mobility ( *p* < 0.001) and work/productivity ( *p* < 0.001) were statistically significant for good QoL, and self-care ( *p* < 0.001) and vision ( *p* < 0.001) were statistically significant for poor QoL ( Table 5 ).

**Table 5:** Significance (%) of domains according to SSQOL

Domains	Energy	Family roles	Language	Mobility	Mood	Personality	Self-care	Social roles	Thinking	Upper extremity function	Vision	Work/ productivity
QOL												
Poor*	5.3 ± 3.4	5.8 ± 2.2	10.5 ± 5.2	9.3 ± 3.7	10.2 ± 4.2	5.4 ± 3.0	13.7 ± 5.5	8.5 ± 3.0	5.8 ± 3.4	10.0 ± 4.4	11.0 ± 3.1	4.6 ± 2.1
Good*	5.4 ± 2.1	5.9 ± 1.5	10.1 ± 2.6	11.8 ± 2.7	10.1 ± 2.4	5.4 ± 1.8	12.0 ± 1.9	9.0 ± 2.6	5.6 ± 1.9	11.2 ± 1.9	7.1 ± 1.6	6.4 ± 1.5
P	0.251	0.383	0.796	**< 0.001**	0.582	0.237	**0.003**	0.232	0.329	0.028	**< 0.001**	**< 0.001**

Notes: *SD: Standard Deviation; **SSQOL: poor<147 points (n=60); good >148 points (n=136). Values in bold indicate statistically significant results.

A receiver operating characteristic (ROC) curve was performed to identify a cutoff point for the NIHSS at admission associated with QoL. The patients with scores > 9 were more likely to have poor QoL ( *p* = 0.001) with 62.1% of sensitivity and 69.7% of specificity.

## DISCUSSION

Our study demonstrated that almost one third of first-time ischemic stroke patients had poor QoL for a long-term period after stroke. It was somehow similar to the results of European countries. The main factors associated with poor QoL were mRS, BADL and IADL, hypertension, irregular rehabilitation (including delay of rehabilitation), unemployment (including retirement), and medical complication after stroke (mood, cognition, neurological and clinical disorders). When using a SSQoL, the affected domains were mobility, self-care, work/productivity, and vision.

Previous international studies report 23 to 40% of patients with poor QoL after 6 months of stroke. 5 28 Although our data comes from a developing country, it appears that stroke has been a global target in health policy all over the world. Recent studies have targeted QoL, but it is still missing attention as a rehabilitation point of care. Three previous studies conducted in Brazil (Northeast and Midwest) about QoL in stroke patients found some different domains' impairment including mood, cognition, and functional disabilities, and going deeper into these differences is necessary. They also cited age, sex, depression, need for a daily caregiver, and financial status as determinant variables for this finding. 3 11 14 We conducted our study in the south of Brazil, which has a better socioeconomic and educational status. The differences could bring more information regarding cross-cultural differences in our larger country. However, as we expected, our population is in line with hypertension as the main risk factor for stroke, and better control directly improves the total care. 29 30 31

Functional disabilities measures with BADL and IADL were found to be the predictors of QoL. However, we showed that 55% of patients who could independently perform BADL were still dependent on caregivers for IADL. It is an important finding that brings the necessity to better evaluate the stroke patient beyond mRS and BI scores (BADL) as isolated measurements for QoL. Our hypothesis based on these results directly goes to the disparity in the assessment of the cognitive and complex functions by the BADL. We could identify a cutoff of NIHSS at admission related to a poor QoL. It was the first citation and should be emphasized. Other studies have suggested different cutoff scores to predict poor outcomes; however, they did not correlate with QoL as an endpoint. 2 3 32

Outcomes were also analyzed in our study. We found that irregular rehabilitation, unemployment, and medical complications after stroke were significant variables for predicting poor QoL. These results can be partially explained by the lack of early and continuous rehabilitation programs. We recommend confirming this data, which can be a valiant direction to political health.

Quality of life is better defined when we focus on domains. Our study found that mobility, self-care, and work/productivity were impacted in our patients with poor QoL. Although some of these domains are also demonstrated in functional scales, the SSQoL scale affords a better description of the difficulties that could be a reference point in rehabilitation plans. An inexplicable finding was a vision impairment in patients without corresponding topographical lesions. This has been previously reported, and the possible explanation was a bias of previous problems. 33

Our study has some limitations. All recording of data was performed by the author that was not blinded to the history or medical conditions, but the questionnaire was answered by the patient (PROs). We also know that there are various scales to measure QoL, and we did not apply more than one to compare. On the other hand, the SSQoL scale is specific for stroke patients and is validated in our language (Brazilian Portuguese). Furthermore, as we excluded recurrent stroke patients, a small number from a single center limited the generalizability of our study.

We consider that QoL needs to be prioritized in stroke studies. The importance and diversity of predictors should be analyzed in each country (even in regions) once healthcare policy can apply the results to improve stroke care. Prospective studies should measure QoL before and after implementing rehabilitation strategies to confirm our hypothesis that better and early rehabilitation can improve QoL after stroke. 34

In conclusion, our study shows that ∼ 30% of patients had poor QoL after their first ischemic stroke, which impaired their ability to participate in daily activities and probably interfered with mental health. No regular rehabilitation and not returning to work appeared to be crucial factors related to it. A functional stroke recovery should encompass parameters of QoL. Stroke is undoubtedly the best model to study QoL once it is considered the most debilitating disease worldwide. We suggest including a more extensive approach in the rehabilitation model that considers not only disability assessment but also encompasses QoL domains in stroke survivors.
